# Supplementary material for: Brain-derived neurotrophic factor stimulates the retrograde pathway for axonal autophagy
Source: J Biol Chem. 2022 Nov 3;298(12):102673. doi: 10.1016/j.jbc.2022.102673 (PMC9768381; doi:10.1016/j.jbc.2022.102673)
Supplement: Supporting information [file mmc1.docx]

**Supporting Information:**

**Brain-Derived Neurotrophic Factor stimulates the retrograde pathway for axonal autophagy**

David Kader Sidibe*, Vineet Vinay Kulkarni*, Audrey Dong, Jessica Brandt Herr, Maria Chalokh Vogel, Max Henry Stempel, and Sandra Maday

*Co-first authors

Affiliations:

Department of Neuroscience, Perelman School of Medicine at the University of Pennsylvania, Philadelphia, PA, 19104, USA

Correspondence: [smaday@pennmedicine.upenn.edu](mailto:smaday@pennmedicine.upenn.edu)

**Movie S1.** **BDNF-Qdots label TrkB-positive signaling endosomes.** Movie of the motility of TrkB-GFP (top panel) and BDNF-Qdots (bottom panel) in the axon of a primary wild type cortical neuron after a short-term incubation with BDNF-Qdots. Retrograde is to the right. In the BDNF-Qdot channel, an outline of the axon is drawn in white. A yellow line is used to separate the TrkB-GFP movie from the BDNF-Qdot movie. Frames were taken once every 2 seconds. The video is played 30X real time. Bar, 3 µm. A kymograph generated from this video is shown in Figure 4.

**Movie S2. TrkB-positive signaling endosomes are not labeled with Qdots alone.** Movie of the motility of TrkB-GFP (top panel) and Qdots (bottom panel) in the axon of a primary wild type cortical neuron after a short-term incubation with Qdots. Retrograde is to the right. In the Qdot channel, an outline of the axon is drawn in white. A yellow line is used to separate the TrkB-GFP movie from the Qdot movie. Frames were taken once every 2 seconds. The video is played 30X real time. Bar, 3 µm. A kymograph generated from this video is shown in Figure 4.

**Movie S3. Short term incubation with BDNF-Qdots does not label the vast majority of GFP-LC3-positive autophagosomes.** Movie of GFP-LC3 (top panel) and BDNF-Qdot (bottom panel) motility in the axon of a primary cortical neuron after a short-term treatment with BDNF-Qdots. Retrograde is to the right. In the BDNF-Qdot channel, an outline of the axon is drawn in white. A yellow line is used to separate the GFP-LC3 movie from the BDNF-Qdot movie. Frames were taken once every 2 seconds. The video is played 30X real time. Bar, 3 µm. A kymograph generated from this video is shown in Figure 5.

**Movie S4. Overnight incubation with BDNF-Qdots does not label GFP-LC3-positive autophagosomes.** Movie of GFP-LC3 (top panel) and BDNF-Qdot (bottom panel) motility in the axon of a primary cortical neuron after overnight treatment with BDNF-Qdots. Retrograde is to the right. In the BDNF-Qdot channel, an outline of the axon is drawn in white. A yellow line is used to separate the GFP-LC3 movie from the BDNF-Qdot movie Frames were taken once every 2 seconds. The video is played 30X real time. Bar, 3 µm. A kymograph generated from this video is shown in Figure 5.
